# How did investigations into spontaneous human combustion influence alcohol medicine? An examination of the medical and literary discussions that brought the two together

**DOI:** 10.1111/add.16739

**Published:** 2024-12-22

**Authors:** Iain Smith, Pam Lock

**Affiliations:** ^1^ Consultant in Addiction Psychiatry, Substance Use Service, Stirling FK7 0BS and Honorary Clinical Senior Lecturer University of Glasgow Glasgow UK; ^2^ Department of English University of Bristol Bristol UK

**Keywords:** alcohol, drinking studies, fat wick burn, gender, isolated central body combustion, literary, medical humanities, spontaneous human combustion, women

## Abstract

**Background and Aims:**

The presence of sections or chapters on spontaneous human combustion in more than half of the key texts in English on the action of alcohol on the body and mind in the first half of the nineteenth century demonstrates the seriousness with which it was considered. We aimed to chart discussions about the links between spontaneous human combustion and spirit drinking in medical texts and representations in fiction through three key chronological periods from 1804 to 1900.

**Methods:**

A contextual analysis using eighteenth‐ and nineteenth‐century historical, literary and medical sources to chart and reflect on public and medical discourses.

**Findings and Conclusions:**

The development of new theories about the action of alcohol on the body and mind appears to have been influenced by the now‐discredited eighteenth‐ and nineteenth‐century idea that the phenomenon of human combustion, spontaneous or not, was linked to spirit drinking. As an extreme example of the consequences of heavy drinking, spontaneous human combustion was used to underpin early theories on the clinical chemistry of alcohol.

## INTRODUCTION

When Professor Griffith Edwards stepped down as editor of *Addiction* in 2004 he was gifted, by the Society for the Study of Addiction, the first published edition of one of the first medical treatises on the action of alcohol on the body and mind: Thomas Trotter's, *An Essay, Medical, Philosophical, and Chemical on Drunkenness and its Effects on the Human Body* (1804). [1] What might seem surprising to a modern reader is that, in Chapter III, ‘In what Manner Vinous Spirits affect the Body’, there is a substantial section on spontaneous human combustion (SHC). Much of this section was a reproduction of Tilloch's translation of the French physician, Pierre‐Aimé Lair's famous article on SHC in the *Journal de Physique* (1800). Although Edwards dismisses this section as ‘somewhat diffuse’, our research has demonstrated that this particular section was influential on the ensuing reflections about the action of alcohol on the body and mind [2]. As such, the text in Trotter's book is the starting point for this article in which we offer a historiographical account of how so‐called SHC has been viewed in the modern era. We will focus on the nineteenth century, as the crucial period for these developments, but we will inevitably consider earlier accounts as they often form the basis of these discussions.

We contend that the incorporation of SHC as a topic in Trotter's *Essay* places the phenomenon firmly in the field of the origins of alcohol medicine. The appearance of discussions of this phenomenon in so many of the subsequent medical‐scientific publications on alcohol of that century demonstrates a discourse linking spirit drinking to SHC, which gathers momentum in the first half of the nineteenth century. This article is divided into three chronological periods to demonstrate these developments. The contentious nature of this topic stimulated both useful medical discussions and the public imagination. Our article below moves between literary and medical representations of SHC, partly because the doctors in question used literary sources in their explanations, and partly because we are interested in the narratives that formulated and reflected on this phenomenon. This interaction between the literary and the medical was one of the stimuli of our collaboration between a literary scholar with expertise on representations of alcohol in nineteenth‐century fiction and a psychiatrist with expertise in addiction and the history of alcohol medicine.

As William F. Bynum observed in his 1968 article on the history of chronic alcoholism, ‘During the 18th and first half of the 19th centuries’ there were two ‘acute manifestation[s] of alcoholism’, that ‘were rigidly defined’, ‘delirium tremens and spontaneous combustion’, although the ‘concept of chronic alcoholism itself [wa]s much hazier’ [3]. The public understanding of links between drunkenness and SHC is clear from newspaper reports, but also by the casual way in which authors included it in their fiction. Journalist and author William Maginn's grotesque ‘The Suicide’ (1824) published in the famous *Blackwood's Edinburgh Magazine* reflects on a range of possible methods for taking his life, but finally settles on drinking himself ‘into the grave’, asserting that ‘I may have the luck to go off in a flash of flame’ [4]. He references American author Brockden Brown, whose novel, *Wieland* (1798), includes a spectacular example of fictional SHC, but the direct link Maginn draws between heavy drinking and SHC must be drawn from medical and popular accounts as this link with alcohol is not made in *Wieland* [5]. Although *delirium tremens* remains a common medical term associated with alcohol dependence (now more formally termed alcohol withdrawal syndrome with delirium), most doctors no longer believe in SHC. Nonetheless, modern understandings of alcohol medicine are partly built on discussions of this strange phenomenon.

## METHODOLOGY

We have examined the publications of 21 of the key doctors writing about alcohol in the nineteenth century and discovered that 11 of 21 include a section on SHC, although there is a definite decline in this trend from the 1870s when it moves into forensic publications rather than those of psychological medicine. As has been observed elsewhere, there is a strong gender bias in the cases described at this time. Of the 57 cases we have so far discovered before 1900, 40 are about women, only 17 men, which are particularly unusual given that male heavy drinking was significantly predominant then as now [6]. There are a few possible theories about this gendered predisposition. Griffith Edwards suggests that ‘we are being invited to see […] the drunkard (especially the woman drunkard) being punished by a spontaneous, personalized, do‐it‐yourself hellfire’ [7]. We have, so far, discovered 10 novels from the eighteenth and nineteenth centuries that include representations of SHC. One of the peculiarities we have noted is that, although the majority of the medical accounts feature women, 9 of 10 of these novels represent men as the victims. We have compared the secondary accounts of SHC with the primary sources, with particular focus on medical publications on drinking and drunkenness to reflect on how the narratives on this relatively rare phenomenon were repeatedly retold to explore the gruesome fascination of physicians and the public with these grotesque stories of habitual drinkers. Therefore, we expand the hitherto under‐developed story of their influence on the beginnings of modern alcohol medicine.

## WHAT IS SHC?

A typical burn pattern is the common thread running through all these cases. The middle section, particularly the torso of the body is usually turned to ash leaving the hands, feet and skull intact. Today, we would describe this pattern as ‘isolated central body combustion’ and see it as a rare, but real phenomenon witnessed by firemen and coroners. The contentious element of definitions of this phenomenon has always been whether or not it is ‘spontaneous’. American physician, Thomas D. Mitchell, a believer in theories of spontaneity, noted in 1822 that they were ‘generally understood’ to be any form of combustion ‘which ensues independently of any thing exterior to the body burnt’ [8]. From the 1840s, new terms developed from ongoing discussions that questioned the spontaneous nature of the phenomenon, such as ‘preternatural combustibility’ [9], ‘increased combustibility’ [10] and ‘increased combustibleness’ [11]. There are early suggestions in these accounts of the wicking concept described in 2011 by Levi‐Faict and Quatrehomme and in 2012 by Koljonen and Kluger, which explains the mechanism of ‘Isolated Central Body Combustion’ [12] as ‘Fat Wick Burns’ [13]. The idea is that if a ‘heat source [chars] and crack[s] the skin’ of an obese torso, it could lead to an escape of adipose tissue, which ignites and acts as a wick to fuel a fire in the centre of the body. This in turn helps explain the pattern of the combustion and its apparent link to obesity and older women given gender differences in fat distribution. It may also help explain the distinctive odour and the colour of the flame often observed.

## THOMAS TROTTER

SHC appears in a treatise on the action of alcohol on the body and mind for the first time in Thomas Trotter's *Essay*, as one of the examples demonstrating his theories about alcohol and hydrogen in the body (1804). As Roy Porter explains in his introduction to Trotter's *Essay*, the original publication in English was described as a second edition of the book because, unusually, Trotter considered his 1788 MD thesis, *De Ebrietate, eiusque effectibus in corpus humanum (On Drunkenness and its effects on the human body*), as the ‘first edition’, although it was written in Latin and about a tenth of the length. [14] There are continuities between the two works, but inevitably new topics are covered in the later *Essay*. Porter sees the main difference between the two works as a broadening of Trotter's analysis from seeing drunkenness as a strictly medical problem to taking into account the idea of habit and habit‐forming drugs within a particular culture: ‘Thus it is fair to say that Trotter began posing drunkenness as a medical problem; only subsequently did he evolve his major insight, the need to investigate the problem of habit and addiction as a whole.’ [14] Although Trotter reflects at length on the physical effects of intoxication and chronic drunkenness, he was one of the first physicians to see drunkenness as ‘a disease of the mind’ [1].

The original MD thesis did not include any mention of SHC; Porter notes this as the main difference in the published version. In his section on SHC, Trotter reproduces a translation of French physician, Pierre Aime Lair's accounts from *Essai sur les combustions humaines, produite par un long abus des liqueurs spiritueuses* (*Essay on human combustion, produced by the long abuse of spirituous liquors*). This is likely to be because Lair's account of the phenomenon was published the same year that this new edition was compiled. This may also explain why it does not appear in the earlier works of the physicians Benjamin Rush and J. C. Lettsom who wrote on alcohol medicine in the late eighteenth century. (Of course, earlier physicians invested in humoral theory saw alcohol as a source of heat in certain circumstances and intoxication could be viewed as too much heat in the brain, but we have found no direct link to SHC to date.) In his *Essai*, Lair sought to demonstrate the link between human combustion and spirit drinking. Trotter reproduces Lair's analysis of the 12 cases of SHC in full with apparent acceptance of almost all its contents. One of the most reproduced parts of Lair's account was the eight common points derived from his analysis:

- The persons who experienced the effects of this combustion, had for a long time made an immoderate use of spirituous liquors.
- The combustion took place only in women.
- These women were far advanced in life.
- Their bodies did not take fire spontaneously, but were burnt by accident.
- The extremities, such as the feet and hands, were generally spared by the fire.
- Water sometimes, instead of extinguishing the flames, which proceeded from the parts on fire, gave them more activity.
- The fire did very little damage, and often spared the combustible objects, which were in contact with the human body at the moment when it was burning.
- The combustion of the bodies left, as a residuum, fat foetid ashes, with an unctuous, stinking and very penetrating soot. [1]

Trotter reproduces these, but does not appear to agree with point IV, and there are suggestions in his preamble that he believes the phenomenon could be spontaneous.

In his *Essay*, Trotter proposes a hypothesis that the ‘large proportion of hydrogen’ in alcohol could explain many of the previously observed physical effects of chronic drunkenness [1]. When considering whether the ‘perspirable matter of drunkards [could be] impregnated with hydrogenous gas’, he proposes that the blood of drunkards might be ‘loaded with hydrogen’, retarding healing and causing diseases of multiple organs. This discussion leads up to his reproduction of the Lair article and he seems to see SHC as an extreme example proving his theories about hydrogen. He immediately prefaces Lair's article with three interesting paragraphs. In the first, he states that human combustion is compatible with his theory that alcohol can cause accumulation of hydrogen in the body. In the second paragraph, he considers that Alexander the Great's body was slow to decompose in the hot climate of Egypt because he was a ‘monstrous drunkard’ and that an accumulation of hydrogen may have preserved his body through a ‘slighter combustion, that might in a manner toast [the body] without burning’ [1]. The third paragraph draws on Shakespeare's well‐known drunkard, Falstaff and his descriptions of his companion Bardolph's nose, glowing in the dark. Trotter reflects that ‘the face of particular drunkards […] appears […] like a burning coal’, implying that there is latent fire in these ‘particular’ drunkards. Trotter's application of these historical and literary examples to his explanation is a common feature of medical treatises of this time to illustrate complex medical ideas at a time when disciplinary boundaries were more permeable. The descriptions of the remains of the older female victims are particularly grotesque and seem to match the taste for the gothic at this time, which typically featured older women as evil and drunken. The repetition of these stories demonstrates a grim fascination with their gruesome destruction and an interest in titillating their audiences with the details. We know that the audience for these books was broad partly from newspaper reviews and adverts in popular and (later) temperance papers and also from the libraries of well‐known authors. In the following sections, we discuss Robert Macnish (whose work was owned and admired by Patrick Brontë, father to the Brontë sisters), James Miller (whose work on alcohol was owned by both Charles Dickens and Wilkie Collins) and W.B. Carpenter (whose work on physiology was admired by George Eliot).

## 1820S—ARGUMENTS FOR AND AGAINST

Thomas Mitchell, writing in *The American Medical Recorder* in 1822, develops on Trotter's chemical theories, defending him from the ‘ingenious Lair’ who ‘could not believe that spontaneous combustion could have place in the human body’ [8]. Mitchell argues that: ‘The fatty matter of the human body, distributed throughout the system, is combustible, and gases are confessedly formed in the human body that are of a combustible nature; and why, then, should it seem strange that the human body should be a subject of spontaneous combustion?’ [8]. He subsequently evidences this argument with a case from Westminster Hospital in which a man died after drinking ‘a quart of gin for a wager’ [8]. On examination, they discovered a ‘limpid fluid, distinctly impregnated with gin, both to the sense of smell and taste, and even to the test of inflammability’ [8]. He, then, uses this account to justify the possibility of SHC, arguing that, ‘it would not be surprising to hear of drunkards being blown into the air in consequence of the explosion of combustible gases formed in their systems’ [8].

Physician J.A. Paris and legal writer J.S.M. Fonblanque wrote a summary of contemporary theories on ‘human combustion’ in their *Medical Jurisprudence* (1823) [15]. Oddly, Paris and Fonblanque mention Trotter and Lair separately, without referring to Trotter's reproduction of or reflection on Lair's article. The list they produce of features of the phenomenon seems to be an overview, in some places more concise, of the Tilloch translation that appeared in Trotter rather than the French original, based on the choices of language and particularly the changes made. However, they disagree with Trotter's theories about chemical reactions in the body and instead affirm that the ‘body has not burned *spontaneously*’ and ‘required for its inflammation the contact or approach of some burning body or that of electric matter’. [15] Interestingly, they mention an early theory by Maffei that ‘lightning is sometimes excited in us, and destroys us’, which explains their aside about ‘electric matter’ in their affirmation. [15].

Coroner, Dr Gavin Thurston claims in a letter to the British Medical Journal (BMJ) in 1938 that the SHC scenes in Captain Marryatt's novel *Jacob Faithful* (1831), which describes the narrator's mother's death, bears a close similarity to those in Paris and Fonblanque's account of SHC. [16] However, Thurston's conclusions do not take into account that Paris and Fonblanque's list is a concision of Lair's original list in Trotter, in which they simply conflate points 2 and 3 of Lair's list, and discards Lair's seventh point about water sometimes exciting the flames. This makes it difficult to establish Marryat's source with any confidence. However, it is clear from the descriptions of the ‘unctuous pitchy cinder’ residue left by the mother's death that Marryat had researched the medical accounts of the phenomenon. [17] Her corpulence and habitual drunkenness demonstrate his understanding of the proposed links between gender, corpulence and regular spirit drinking.

First published in the same year as Paris and Fonblanque, Beck's *Elements of Medical Jurisprudence* (1823) [18] is most famous in the history of SHC as the key text in Dickens's defence of Krook's death by SHC in *Bleak House* (1852) against criticism by G.H. Lewes (described below). Interestingly, although Dickens cites Beck's as proof of his belief in SHC, their footnotes deny that the phenomenon was spontaneous: ‘Probably the term spontaneous is not strictly accurate, since we always find some burning body mentioned as exciting the phenomenon in question. I have adopted it, because of its general use, and also because the term human combustion, which it has been proposed to substitute in its place, appears too indefinite’. [18].

Author and physician, Robert Macnish's *Anatomy of Drunkenness* (1827) [19] was the next popular medical text to focus solely on alcohol. He was already well‐known in popular and medical circles for his book on sleep and his connections to the early phrenological movement. The aim of his monograph was to outline the causes, effects and possible treatments for acute and chronic drunkenness and he includes a chapter on ‘Spontaneous Combustion of Drunkards’. Although Macnish does not mention Trotter in this chapter, only references Tilloch's version of Lair's work, we know that he was familiar with Trotter's writings from his discussions of the *Essay* in other chapters of *Anatomy*. Macnish's discussions of hydrogen, which were originated by and peculiar to Trotter's approach, therefore, seems to be an unattributed response to Trotter's ideas on this. He starts the chapter with a sentence on hydrogen, demonstrating that Trotter is likely to be the inspiration for the chapter and at the forefront of his mind: ‘Whether such a quantity of hydrogen may accumulate in the bodies of drunkards as to sustain combustion, I profess myself totally unable to determine’ [19]. He goes on to complain that the subject ‘has never been satisfactorily investigated’ and ‘notwithstanding the cases brought forward in support of the doctrine’ [19].

Although Griffith Edwards claims that Macnish accepted SHC, our reading of *Anatomy of Drunkenness* demonstrates that Macnish was very sceptical [7]. Macnish states in the first paragraph that ‘the general opinion seems to be, that the whole is fable’ [19]. He, then, ponders the number and range of expert writers who have ‘given their testimony in support of such facts’, but cannot shake the conviction that ‘their relators have been led into an unintentional misrepresentation’ [19]. Macnish does not even believe that a drunkard, on catching fire, would be more ‘inflammable than […] the soberest person’. [19] He argues that ‘[i]t seems probable that to the effects of a stroke of lightning are we to impute many of the cases in question’ [19]. He does admit that if anyone could prove that phosphuretted hydrogen builds up in the body as suggested, it could be ‘sufficiently potent to account for the burning’, but dismisses all evidence to date as ‘vague report’, claiming that chemists are ‘indifferent physiologists’ who have neglected to understand the post‐mortem nature of the processes of decomposition, which could make this possible. [19] He concludes that these accounts ‘wear, unquestionably, the aspect of fiction’ [19]. Nonetheless, he gives a detailed account of several cases so the reader can ‘judge for himself’ demonstrating the seemingly irresistible urge for physicians to tell these grotesque stories. He goes on to say that even the less dramatic accounts of people ‘blown up in consequence of their breath or eructations catching fire’ are untrue and come from American sources claiming that they have a ‘propensity for the marvellous’ [19]. This criticism of American sources is likely to be influenced by two publications already mentioned, Mitchell's ‘Remarks’ and Brockden Brown's novel, *Wieland* (1798). The latter was widely read in Britain and includes a ‘marvellous’ account of SHC. Brown describes in detail the death of the narrator's father, who is discovered on fire, but still alive in a stone temple near their home. At first, they think ‘his clothes had been removed and only discover later that they were “reduced to ashes”’ although ‘[h]is slippers and his hair were untouched’ [5]. The author's descriptions, although gothic and grotesque, match the early medical accounts carefully, the intact clothing on the head and feet suggestive of the burn pattern described in extant cases of SHC.

Likewise, the medical accounts not only ‘wear […] the aspect of fiction’ or ‘fable’, but often have the feel of fiction themselves. For example, there is a fictional feel to the story of the death of Mary Clues (or Chies) by SHC in Coventry in 1773. This account, purportedly from a letter from local surgeon, Mr Wilmer, is repeated by Lair, Tilloch, Trotter, Macnish and Wilson. Wilmer's account, although loaded with medical details, is told in the style of a contemporary novel. After describing her as ‘addicted to intoxication’, he explains that her ‘propensity to this vice had increased after the death of her husband [until] scarcely a day had passed in the course of which she did not drink at least half a pint of rum or anniseed‐water’ like a narrator [1]. He goes on to describe her discovery in dramatic terms: ‘smoke was seen issuing through the window, and the door being speedily broke open, some flames which were in the room were soon extinguished’ [1]. The majority of these physicians repeat Lair's account verbatim, seemingly relishing the detail. Wilson shortens it (citing Tilloch's translation of Lair), but in doing so, focuses in on the grotesqueness of her drunkenness with more extreme language. In Wilson's version, she is ‘grossly addicted to drink’, not just ‘addicted to intoxication’ and she ‘swallowed from half a pint to a quart of rum’ every day rather than ‘at least half a pint’. These small alterations embroider the already grotesque story. These gothic choices of language and the atmosphere they create demonstrate the influence of contemporary fiction on the writing of these physicians. This awareness of readership becomes clearer in publication practices later in the century. For example, W.B. Carpenter's prize‐winning *On the Use and Abuse of Alcohol Liquors in Health and Disease* was simplified as a ‘new’ edition for the British publisher, Henry G. Bohn, entitled *The Physiology of Temperance and Total Abstinence*. *Being an Examination of the Effects of the Excessive, Moderate and Occasional Use of Alcoholic Liquors on the Healthy Human System* in which he simplified the language and explanations for a general audience (the American edition had explanatory editorial notes for the same purpose) as well as substituting examples of ‘more general interest’ for the public. Given that these texts were also intended for a wide readership, it is likely that these writers were deliberately making their medical case studies more accessible and enjoyable for their public through literary techniques.

## 1850S—W.B. CARPENTER, G.H. LEWES AND DICKENS

Gavin Thurston's letter to the BMJ suggests that ‘one can picture temperance fanatics making much of the phenomenon of spontaneous combustion’ [16]. Unbeknownst to Thurston, his imagined attribution of this argument to temperance campaigners is evidenced in the literature of that movement. The well‐respected physiologist and temperance advocate, W.B. Carpenter, mentions SHC in several of his writings on alcohol. In a footnote in an early work, *Principles of General and Comparative Physiology* (1839), he defends the ‘well‐authenticated instances in which combustion has commenced without the proximity of an ignited body’ and reflects on the possible links between phosphorus in the body and phosphorescence of bodies after death, which seem to respond to Macnish's challenge to theories on the presence of phosphuretted hydrogen in the bodies of drunkards. Although he seems to have changed his mind about the possibility that human combustion of this sort could be spontaneous by the time he wrote *On the Use and Abuse of Alcoholic Liquors* (1850)*,* he did continue to expand on theories about the presence of phosphuretted hydrogen in heavy drinkers, claiming that ‘the *breath* of drunkards has been sometimes observed to be luminous, as if it contained the vapor of phosphorus or of some of its compounds’ [20]. In his notes, he argues that although the ‘phenomenon termed “spontaneous combustion” of the human body’ was rare, ‘it should not be passed by in any inquiry into the consequences of such excess’ because it represented an extreme version of the ‘perverted nutrition’ to which he had already attributed ‘various other consequences’. [20] Matthew Osborn's monograph on *delirium tremens*, *Rum Maniacs* (2014), argues that ‘[p]ublicizing delirium tremens and the spontaneous combustions of drunkards was part of physicians’ larger campaign to share knowledge that could persuade those already temperate to remain so’ and ‘became subjects of popular speculations and proliferated in ways physicians had not intended’ [21]. This practice was less common in British medical publications, and predominantly appears in the writings of temperance doctors such as W.B. Carpenter.

Although Charles Dickens wrote vitriolically against the temperance ideas that W.B. Carpenter's work supported, he did agree with Carpenter that human combustion might be linked to heavy drinking. In *Bleak House* (1852), Dickens wrote a grotesque and dramatic death scene for the desiccated and drunken landlord, Krook. George Eliot's partner, G.H. Lewes, the well‐known critic and scientific journalist, had public disagreements with both Dickens and Carpenter during this period. He tore into Dickens about his depiction of SHC and systematically destroyed Carpenter's arguments about teetotalism. As Gordon S. Haight has explored, Lewes accused Dickens of ‘overstepping the limits of Fiction, and giving currency to a vulgar error’ in response to Krook's death scenes in *Bleak House* and declares spontaneous combustion to be ‘absolutely impossible, according to all known laws of combustion, and to the constitution of the human body’ using the flaming of a Christmas pudding as proof (‘the alcohol will burn, but *not* the raisins’) [22]. Unusually, Dickens defended himself within his fiction in the next published part of the novel by adding an unplanned coroner's inquest in the next chapter that named his real‐life sources. In this report, he mentions real accounts of SHC in the ‘sixth volume of the Philosophical Transactions; and also of a book not quite unknown on English medical jurisprudence’ [23]. The ‘Philosophical Transactions’ is probably Tilloch's translation of Lair and the ‘English medical jurisprudence’ he mentions is Beck's *Elements of Medical Jurisprudence*. Many of the descriptions of the discovery of Krook's remains are taken directly from these accounts. The ‘log of wood’ analogy for a burned limb echoes an account of the case of ‘Grace Pett’, for example, which is taken directly from Tilloch's translation of Lair (the wording is extremely similar), and the greasy ashes match Captain Marryat's descriptions in *Jacob Faithful* (1831) [17], which Lewes claims as Dickens's source, but also match Lair's description of an ‘unctuous, stinking, and very penetrating soot’ [1]. Their public fight not only popularised the mythology around SHC and its links to spirit drinking once more, but may have influenced the continuation of these discussions in the medical treatises of the 1850s.

## 1870S–1890S—THE SHIFT TO MEDICAL JURISPRUDENCE

In the later nineteenth century, there was a move to more identifiable specialisms including medical jurisprudence. As a result, discussion on SHC became confined to specialist publications in this area. Physicians such as Ogston (1870) [10] and Hava (1894) [11], further questioned the ‘spontaneous’ element of the theory and instead suggested the person may have died before the combustion. Hava argued that ‘the abuse of alcoholic liquors had been suspected, and that the accumulation of alcohol was theoretically, but not practically, its cause’, challenging the idea that excess consumption could cause this ‘increased combustibility’ [11]. However, others still linked these events to alcohol by suggesting that the corpse of a drinker was more prone to combustion. Ogston proposes that ‘*the weight of authority is in favour of spontaneous ignition*, or, at least, of increased combustibility’ (original emphasis) and demonstrates his conviction that alcohol could be present in the body in large quantities with a case observed by his father in which ‘there existed so much alcohol in the body that the serum in the ventricles of the brain caught fire and burned on the approach of a lighted match’ [10]. Again, there is resonance of this shift in fiction. In *Doctor Pascal* (1893), French author Emile Zola [24] includes a grotesque account of the narrator's brother succumbing to ‘increased combustibility’ in a manner similar to Hava's descriptions of a ‘wick’ effect in burning bodies where the ‘melted fat [acted] as combustible matter, oxide of carbon as inflammable gas’, like the more modern notion of ‘fat wick burns’ [11]. The narrator describes the victim with impatience as a heavy drinker, ‘in a sort of coma’ [24]. The source of the flame is ‘his pipe [which] had fallen on his knees’. At first, she watches quietly: ‘she could see the bare flesh of his thigh—red flesh, from which a little blue flame was arising’ [24]. When she tries to wake him, she discovers the ‘skin of the thigh cracked, and the fat commenced to melt […] feeding the flame which was beginning to lick his stomach’ [24]. She blames alcohol: ‘For years past he had been saturating himself with spirit of the strongest, most inflammable kind’ [24]. She can see from his breathing that he is still alive, but unnervingly calm, she leaves quietly rather than acting further to save him, stopping only to pick up her gloves. The careful description of the action of the fat and the flame matches medical accounts like Hava's. The forensic pathologist was by this point the person required to find an explanation for these rare but distinctive cases. In his *A Treatise on Medical Jurisprudence* (1901), Vivian Poore [25] attributes SHC to post‐mortem changes producing gases that might indeed combust under certain circumstances; he makes comparison to haystacks that are occasionally known to ignite.

Toward the end of the century, *delirium tremens* continued to be central to discussions of habitual drunkenness, but SHC was no longer to be found in the substantial accounts of ‘alcoholism’ as it was becoming known. Scottish physician Norman Kerr, who helped found the Society for the Study and Cure of Inebriety, makes no mention of SHC in his significant textbook, *Inebriety* (1888) [26]. Similarly, the French professor and temperance campaigner, Maurice Legrain, who wrote the alcohol‐related entries in Tuke's *Dictionary of Psychological Medicine* (1892) [27] makes no mention of SHC in any of his entries.

## CONCLUSIONS

There is something of a gothic fascination in the way that these narratives, focused on the bodies of older, grotesque women, are repeated. The gruesome detail makes the stories more engaging, more entertaining, and therefore, more powerful in both the logical and creative imaginations of the readers. This fascination with the stories of SHC continues with the recent studies mentioned above, popular books such as Randles and Hough's, *Spontaneous Human Combustion* [28] and Bondeson's *A Cabinet of Medical Curiosities* [29] and (usually comedic) televisual versions such as *This is Spinal Tap* (1984), *South Park* (1999) and *Bones* (2009), which reflect the comic versions in fiction of the nineteenth century such as Maginn's ‘The Suicide’ mentioned above and W.E. Ayton's ‘How we got up the Glenmutchkin Railway’ [30]. The re‐telling of these stories, embellished and altered, is reminiscent of the didactic power redolent in the oral tradition; learning is solidified and made real through stories in both fiction and medical narratives. The interaction between fiction and these narratives demonstrates the power of fiction in imagining and reflecting on difficult, nuanced topics.

The development of new theories about the action of alcohol on the body and mind was influenced by the now discredited idea that the phenomenon of human combustion, spontaneous or not, was linked to spirit drinking. Nevertheless, they played an important role in the developments in the nineteenth century. As an extreme example, they helped physicians and chemists in the early developments toward biochemistry to reflect on the possible chemical reactions in the body that might be caused by the addition of alcohol to the human system.

When we set out on this research path, we did not expect that the interplay between the medical literature and the contemporary fiction would be as rich as it turned out to be. This partnership is an example of the value of truly interdisciplinary research practice, in this case unusually between a psychiatrist with expertise in both treating addiction and an MD in the history of alcohol medicine, and a literary scholar in drinking studies with an historicist approach experienced in medical humanities. Reading these nineteenth century texts in parallel together has allowed us to gain an enhanced understanding of the interlinked medical and public discourses on SHC and spirit drinking, which would not have been possible as sole researchers.

## AUTHOR CONTRIBUTIONS

**Iain Smith:** Conceptualization (equal); data curation (equal); formal analysis (equal); methodology (equal); writing—original draft (equal). **Pam Lock:** Conceptualization (equal); data curation (equal); formal analysis (equal); investigation (equal); methodology (equal); resources (equal); writing—original draft (equal).

## DECLARATION OF INTERESTS

None.

## Data Availability

Data sharing not applicable to this article as no datasets were generated or analysed during the current study.
